# Bilateral carotid body tumor case: A novel preoperative management

**DOI:** 10.1016/j.radcr.2021.11.009

**Published:** 2021-12-23

**Authors:** Alberto Moscona-Nissan, Carlos A Saldívar-Rodea, Rocío Enríquez-García, Laura I. Rincón-Ángel, Andrea Navalón Calzada, Alec Seidman-Sorsby, Mayte Cruz-Zermeño

**Affiliations:** a School of Medicine, Universidad Panamericana, Mexico City, Mexico; b Interventional Radiology Department, Hospital General de México Dr. Eduardo Liceaga, Mexico City, Mexico

**Keywords:** Carotid body, paraganglioma, head and neck, embolisation, graft stent, Shamblin

## Abstract

Paragangliomas are rare neuroendocrine neoplasms. The most common form of these tumors in head and neck are non-functional carotid body tumors. These neoplasms may present an extensive growth and compromise vital neurovascular structures in the neck, such as carotid vessels. Carotid body tumors usually present clinically as painless neck masses and occur most frequently in adults averaging 45 to 50 years, being the majority of these tumors unilateral and only 5% of all cases bilateral. The main treatment for carotid body paragangliomas is surgical resection, which can be extremely challenging due to tumor hypervascularity and significant blood loss.

We present a bilateral carotid body tumor case in a 61-year-old woman who presented due to a pulsatile and painless mass in the right carotid region of the neck of 1-year of evolution. The tumor was found encasing the external carotid artery and classified as Shamblin II. A novel approach for preoperative management was performed, placing a covered graft-stent in the right common and proximal (C1) internal carotid arteries in order to splint and provide structural protection for carotid vessels during surgical resection and temporarily reduce blood flow of the carotid body tumor.

## Introduction

Carotid bodies are chemoreceptors located in the carotid arteries adventitia, near the bifurcation level. They are the largest paranglia of the head and neck, measuring 3 to 5 mm and the most important source of catecholamines during the fetal period until development of the adrenal gland. Carotid bodies are mainly irrigated by the ascending pharyngeal artery, a branch of external carotid artery and innervated through Hering's nerve by the glossopharyngeal nerve. Carotid bodies are the organs receiving the richest blood supply according to their weight. They function as chemoreceptors, regulating blood oxygen concentration by sensing systemic changes in pH, carbon dioxide and oxygen [1].

Carotid bodies are part of the paraganglia system, being a carotid body tumor a paraganglioma, which is a neuroendocrine neoplasm. Carotid body tumors (CBTs) present an extensive growth and may compromise and encase vital neurovascular structures in the neck, such as the external and internal carotid artery, making surgical resection extremely challenging [2]. Although CBTs are neuroendocrine neoplasms, they rarely produce catecholamines and most of these tumors are benign. Although infrequent, CBTs are the most common paraganglioma of the head and neck, with an approximate incidence of less than 1 case in 30000 people, especially affecting women with an average age of 43 years, being the female to male ratio of 8:1 [3].

## Case

A 61-year-old woman presented with a chief complaint of a neck mass that persisted 1 year, with no other associated symptoms. On physical examination, a pulsatile and painless mass was palpated in the right carotid region of the neck. On palpation, a tumor with regular borders, soft consistency, unadhered to deep compartments and movable when swallowing was found. No temperature or color changes were present. Vital signs were found within normal limits.

**Fig. 1:**
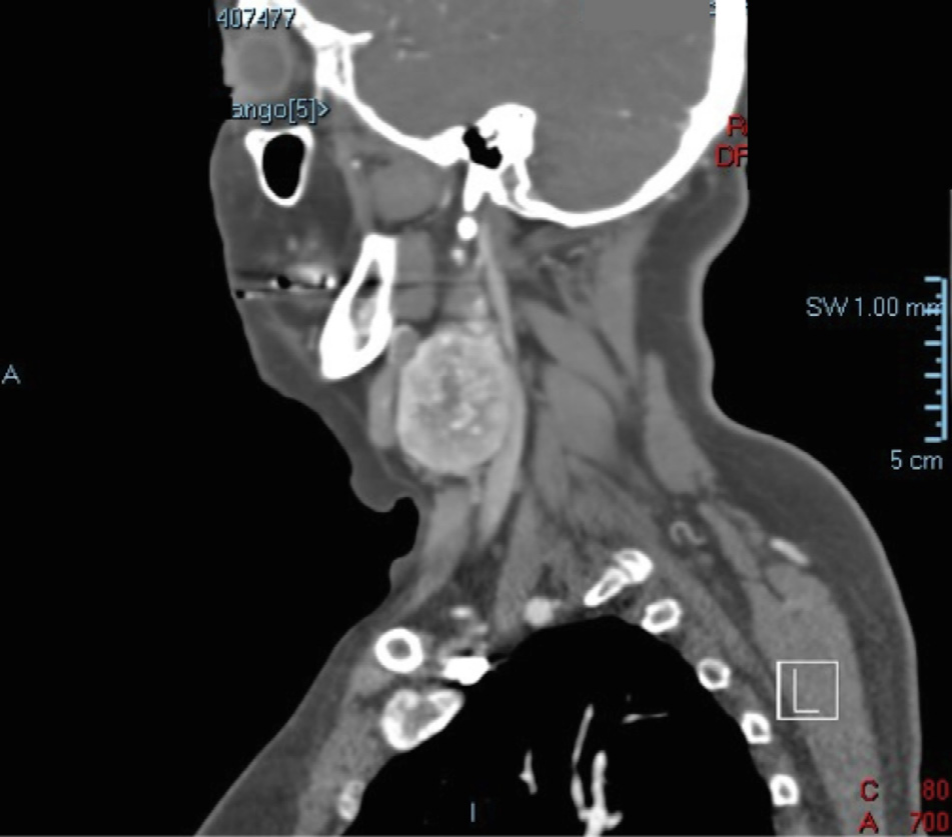
Contrasted supra-aortic trunk angiotomography, sagittal cut

**Fig. 2:**
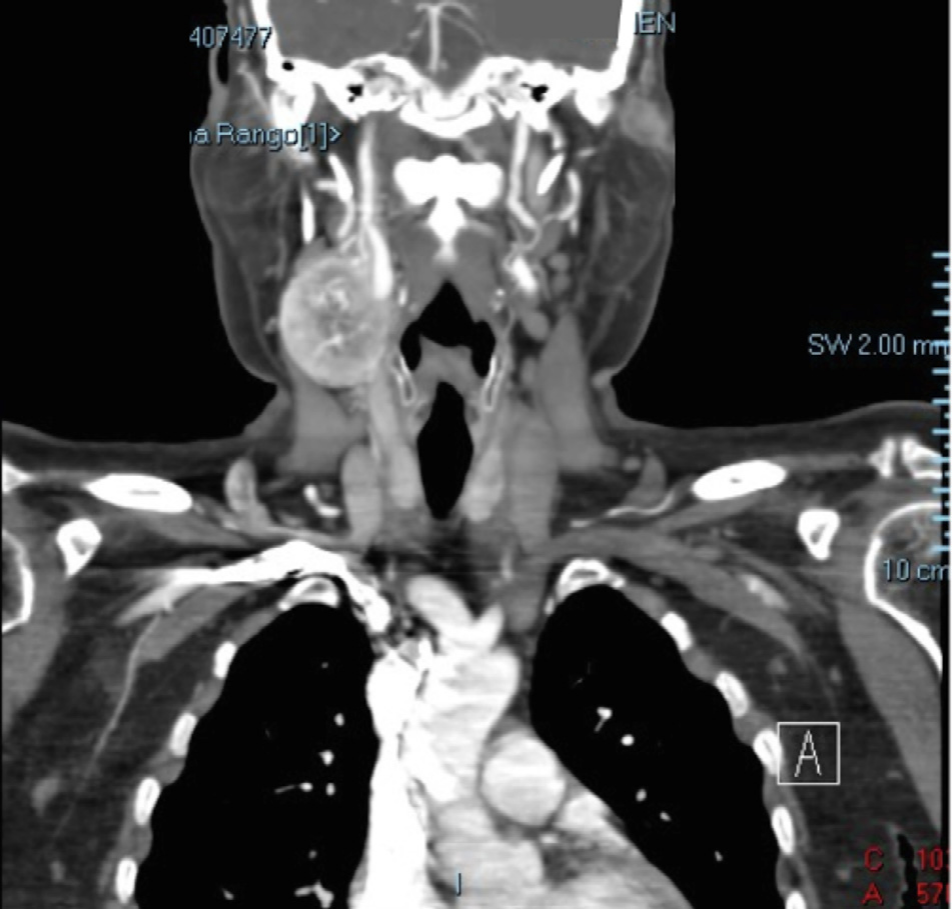
Contrasted supra-aortic trunk angiotomography, coronal cut

A supra-aortic trunk angiotomography, revealed an heterogeneous nodular and hypervascular solid mass of 4.2 × 3.6 × 3.4 cm in the right carotid space (Figs. 1, 2 and3). The mass superior limit was the posterior border of the gonial angle and extended caudally to C5 height. The mass was found encasing the proximal portion of the external right carotid artery (Fig. 4). Adjacent structures to the mass were submandibular glands (anterior), pharyngomaxillary space (anteromedial), pharyngeal mucosa (medial), right jugular vein (posterior) and sternocleidomastoid muscle (posteromedial).

**Fig. 3:**
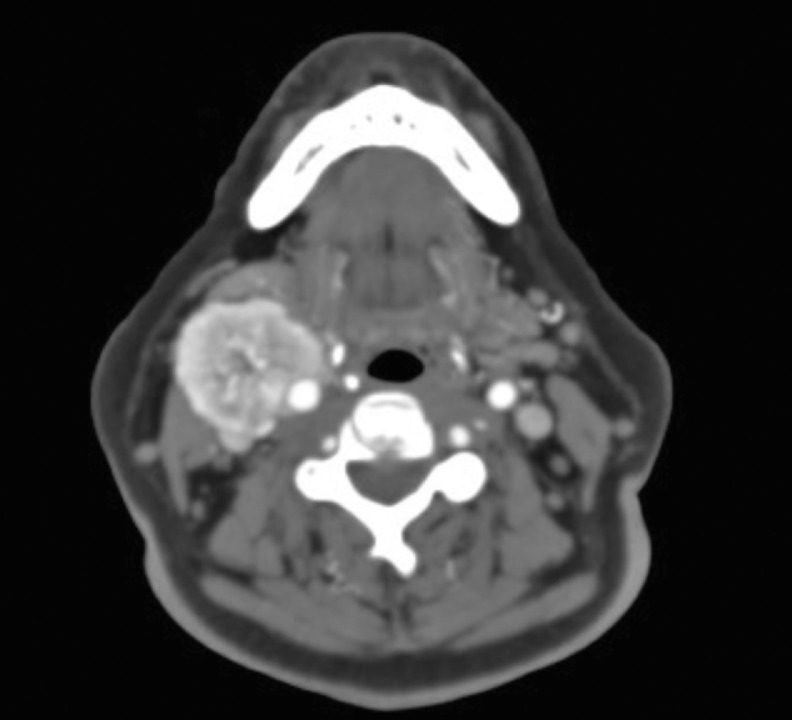
Contrasted supra-aortic trunk angiotomography, axial cut at common carotid artery level

**Fig. 4:**
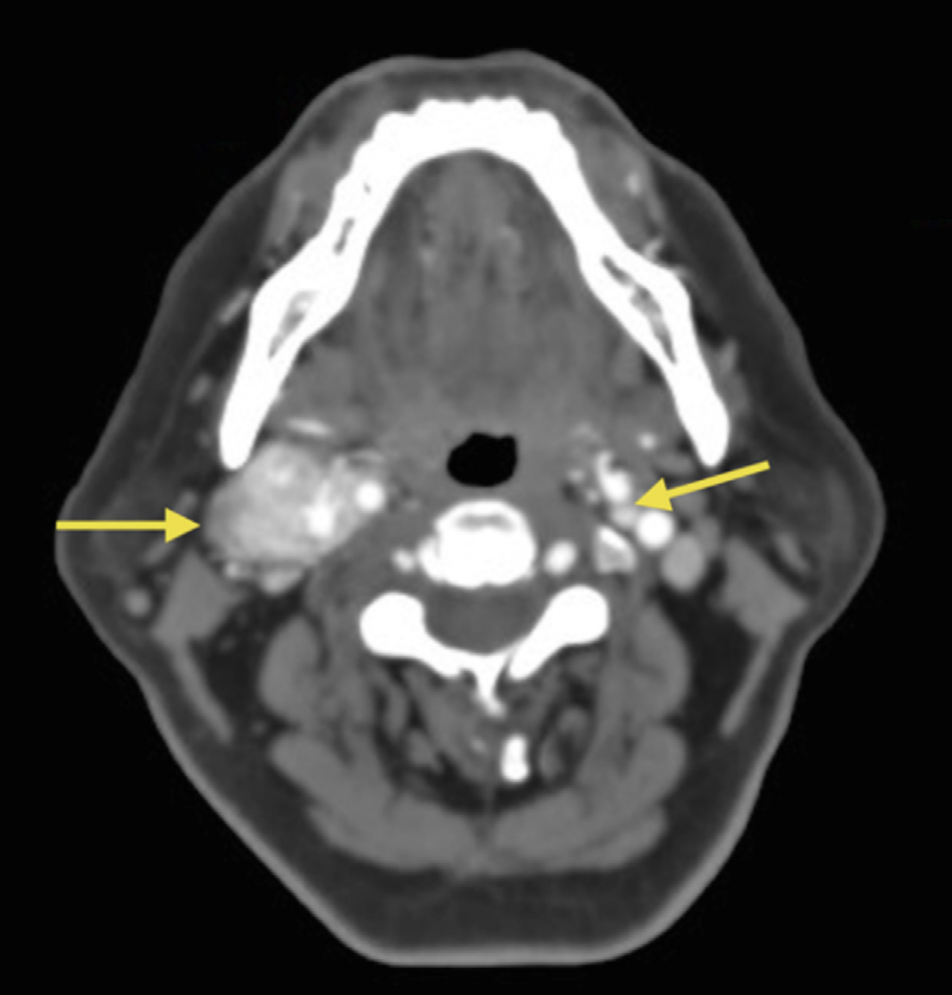
Contrasted supra-aortic trunk angiotomography. An axial cut after common carotid bifurcation, showing right and left carotid body tumors

On the left side of the neck, a bilateral carotid body tumor of 6.4 × 5.8 mm was found in the carotid bifurcation (Fig. 4). Additionally, vein collaterality and increased caliber in transverse cervical veins crossing the trapezius muscle was observed due to inability to drain into the external jugular vein. No signs of thrombosis were found.

A right carotid body tumor encasing the proximal external carotid artery was diagnosed and classified as Shamblin II (Fig. 4), due to partial or focal adventitial infiltration of the carotid vessels. Initial therapeutic management included an extensive valoration of the patient's clinical history and laboratory tests, finding no absolute contraindications for the collocation of a preoperative graft-stent. The procedure required access through the right common femoral artery. Under fluoroscopic control, a hydrophilic guide wire 0.035″ and 5Fr HeadHunter catheter were introduced in order to canalize both common carotid arteries and right internal carotid artery in C1 segment.

**Fig. 5:**
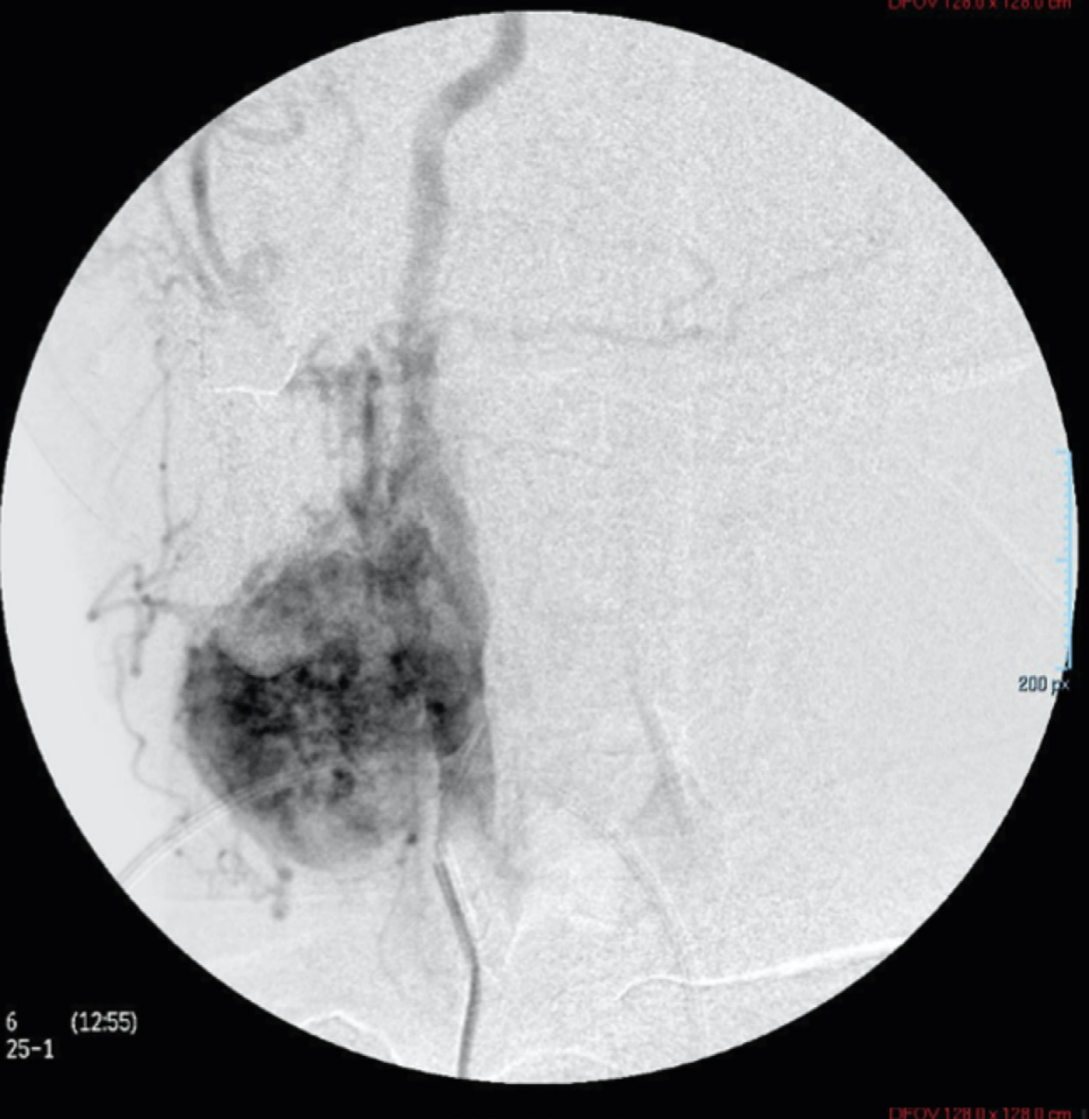
Angiography of right common carotid artery showing abnormal filling in relation to carotid body tumor

A right common carotid artery angiography was performed (Figs. 5 and6), revealing right opacification of the carotid body tumor, which surrounded partially and laterally the right external carotid artery and displaced medially the C1 segment of the right internal carotid artery. The aortic arch, both common carotid arteries and left external and internal carotid arteries were found with no alterations.

**Fig. 6:**
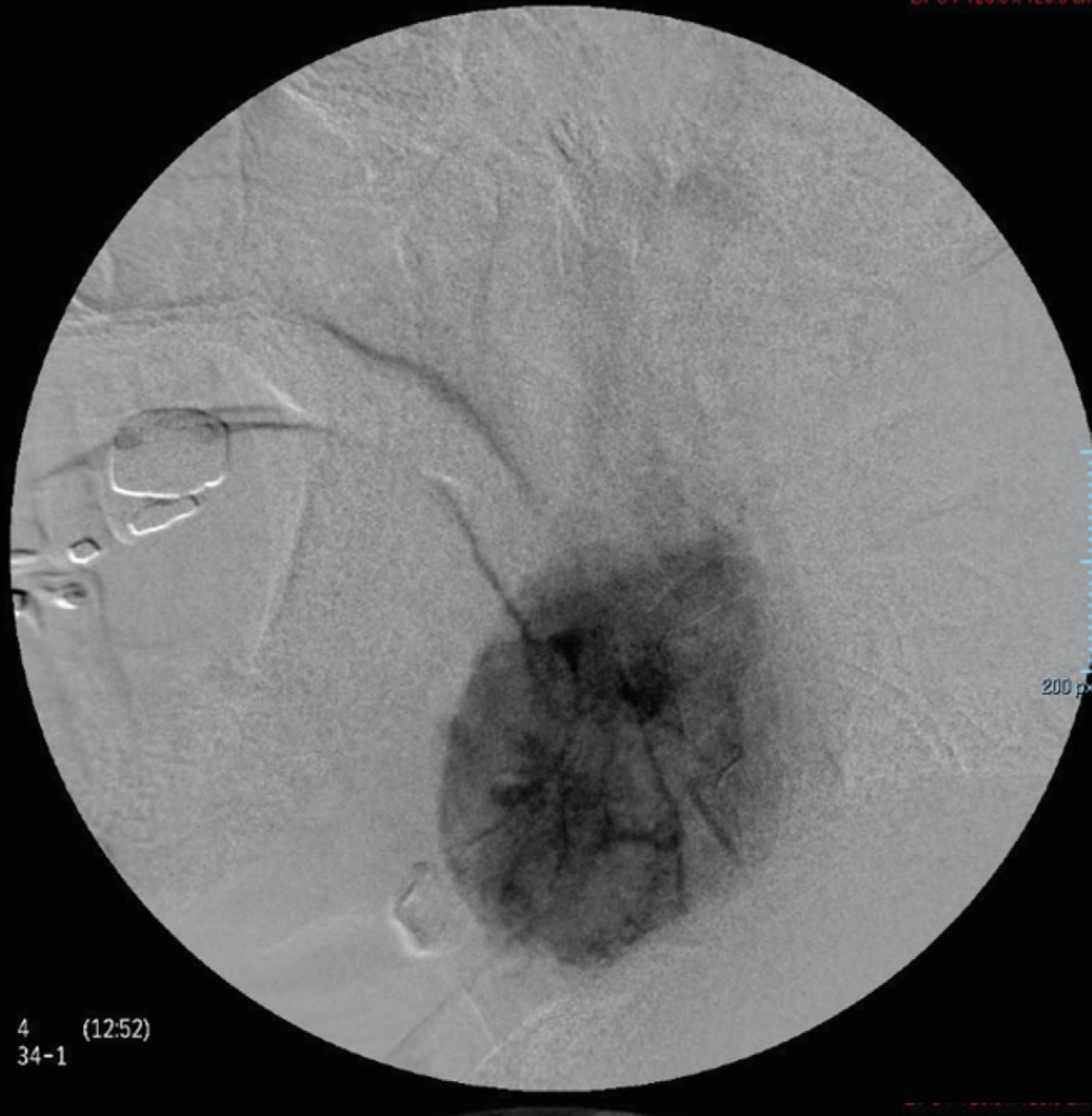
Angiography of right common carotid artery showing abnormal filling in relation to carotid body tumor

Provocative test occlusion or Matas test was performed by compressing the right common carotid artery in order to test the adequacy of the anterior communicating artery, finding inadequate collateral circulation in the present case.

**Fig. 7:**
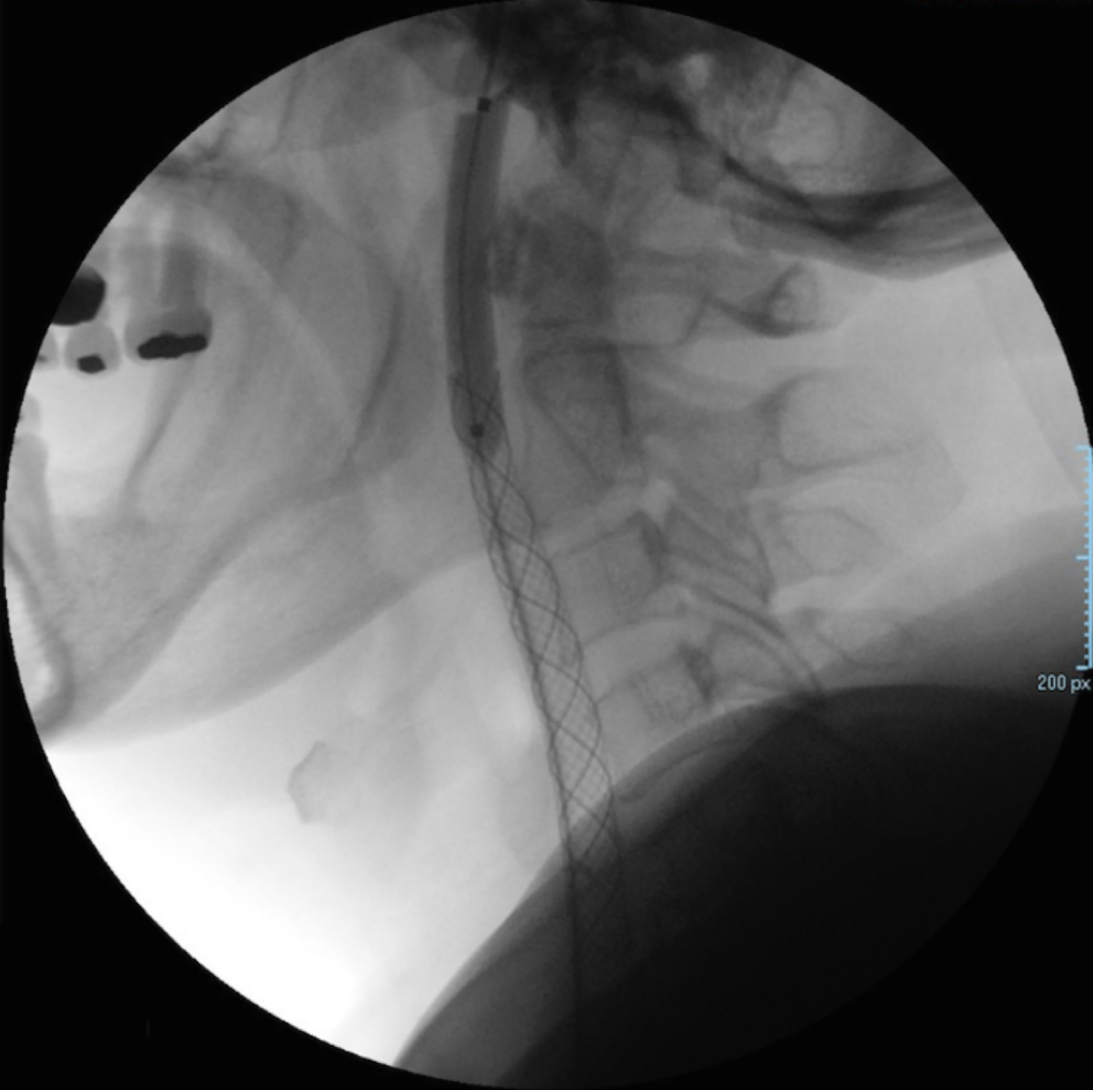
Graft-stent colocation in the right common and internal carotid arteries (C1)

Subsequently, the 5Fr HeadHunter catheter and hydrophilic guide wire 0.035″ were removed, inserting instead an 11Fr sheath introducer in order to place an 8 mm diameter and a 70 mm length polytetrafluoroethylene (PTFE) covered graft-stent in the right common and proximal internal carotid arteries (Fig. 7). Blood flow towards the external carotid artery was blocked temporarily, reducing tumor´s vascularity and size prior to surgical resection (Figs. 7 8, and 9). Due to artery spasm, 6.0 cc of lidocaine and 2.0 cc of nimodipine diluten in physiologic solution were administered. Due to a filling defect and possible dissection, a balloon plasty was performed in the artery segment distal to the stent. Finally, the patient underwent a magnetic resonance imaging (MRI) and a fluid-attenuated inversion recovery (FLAIR) sequence control in order to discard any ischemic lesion, finding no abnormalities. On patient follow-up, no clinical alterations were found in the physical examination, an MRI was performed, revealing no vascular abnormalities.

**Fig. 8:**
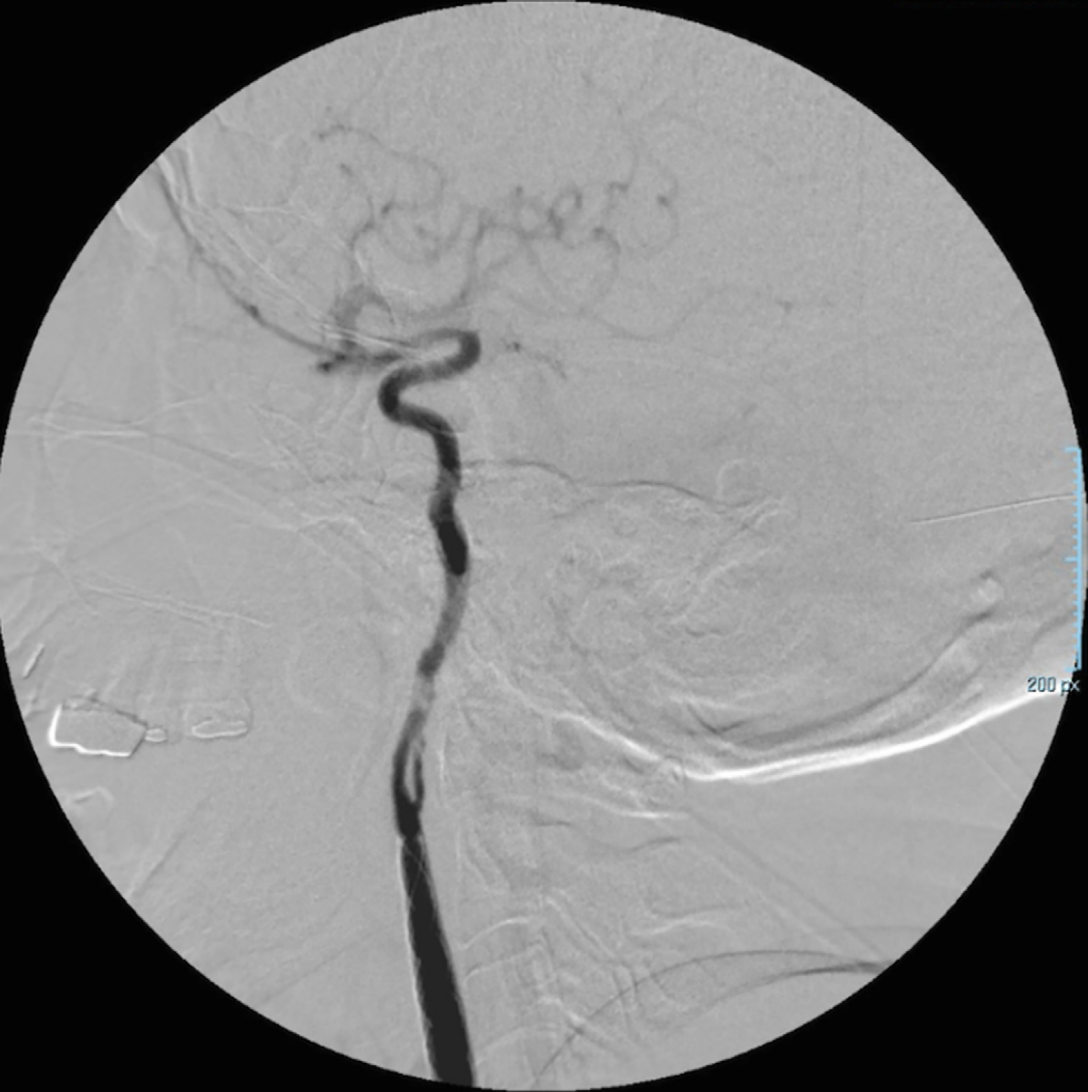
Right internal carotid artery angiography after sent colocation showing probable flap of the *tunica intima* in C1 segment

**Fig. 9:**
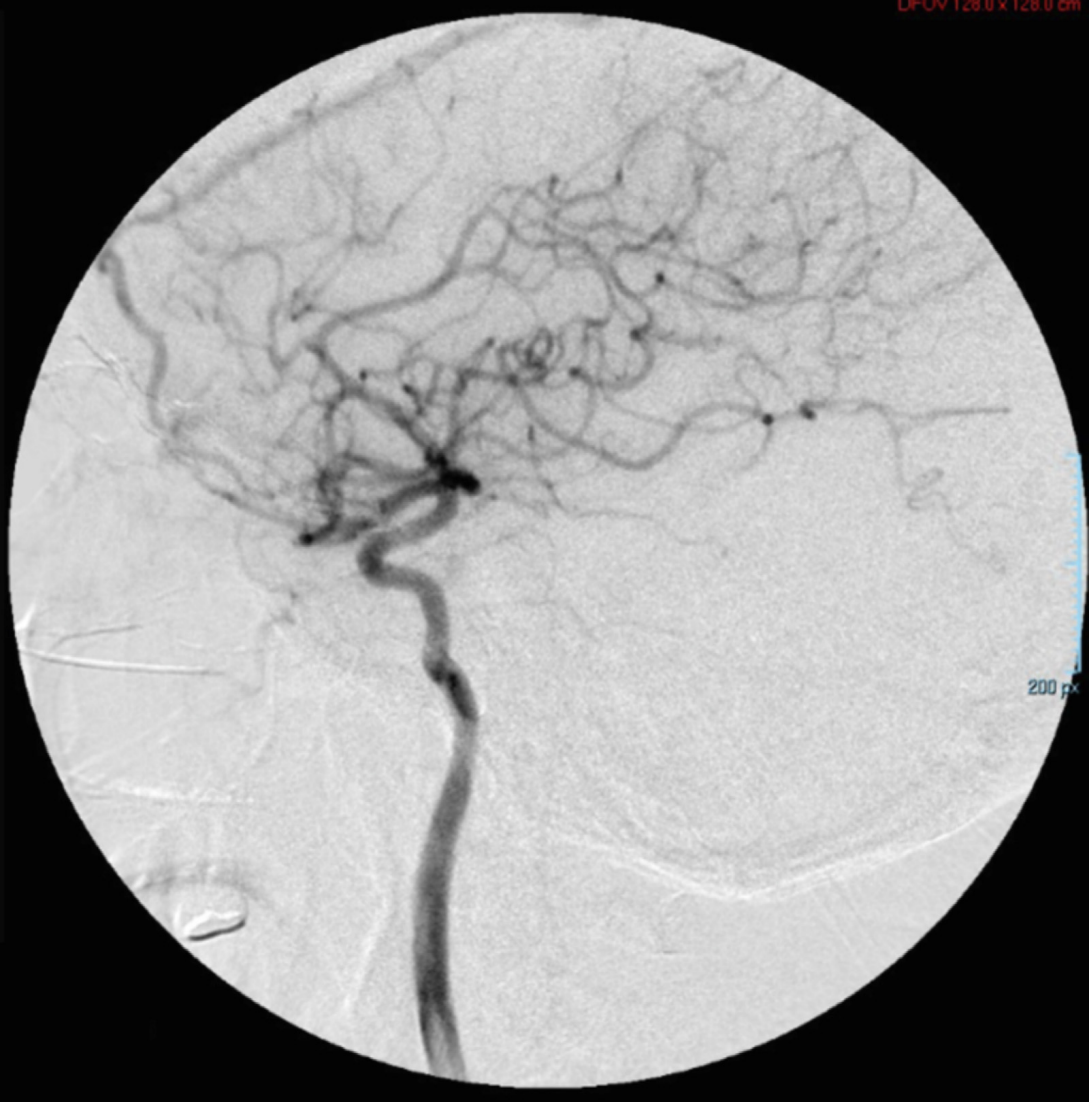
Right internal carotid artery angiography after balloon plasty of the flap, showing adequate blood flow towards the cerebral circulation

## Discussion

### Epidemiology and etiology

Paragangliomas are rare neoplasms, usually detected in women with an average age of 43 years, with a female to male ratio around 8:1. The exact incidence is largely unknown because the clinical patterns are commonly described in conjunction with pheochromocytomas. The combined estimated annual incidence is approximately 0.8 per 100,000 person-years in the United States. The incidence of CBTs is less than 1 in 30,000, representing nearly 65% of head and neck paragangliomas [4], [5], [6]. CBTs occur most frequently in adults averaging 45 to 50 years, being the majority of these tumors unilateral (95%). Bilateral CBTs represent approximately 5% of all cases and are usually described in familial forms [7,8].

There are three main etiologies of CBTs, which are sporadic, familial and hyperplastic. The sporadic etiology corresponds to 85-90% of the total cases. The familial form represents 10% of the cases, being 32% of familiar CBTs bilateral. The hyperplastic form of carotid body paraganglioma is mainly related to high altitudes and chronic diseases that cause hypoxemia, like Chronic Obstructive Pulmonary Disease (COPD) due to possible chronic stimulation of the carotid body [1].

Although the most common cause in CBTs is sporadic, 30%–50% of cases have a component of an inherited syndrome [5]. Most hereditary paragangliomas, particularly those arising from the head and neck, have been associated with pathogenic variants in which they encode different subunits of the succinate dehydrogenase (SDH) enzyme complex. [5] SDH plays a central role in energy metabolism as both an enzyme of the tricarboxylic acid cycle and as complex II of the mitochondrial respiratory chain, catalyzes the oxidation of succinate to fumarate in the Krebs cycle and couples that with electron transfer to the terminal acceptor ubiquinone in the electron transport chain [9].

Five hereditary syndromes related to SDH mutations have been described, all of them characterized by an autosomal dominant (AD) inheritance pattern with variable penetrance, tumor risk and malignancy rates [5]. SDH gene includes several subunit genes (such as SDHA, SDHB, SDHC, SDHD) and cofactors (SDHAF2) [9].

PGL1 syndrome (SDHD mutations) is the most common type of familial paraganglioma syndrome. The penetrance is about 90% or higher by age 70. Most patients with this mutation develop multiple head and neck paragangliomas, but other extra adrenal tumors have been described as well. The associated risk of malignancy is less than 5% [9].

PGL2 syndrome (SDHAF2 mutations) cancer susceptibility has been associated only with paternal transmission, penetrance reaches 100% by age 50 and barely none of the tumors are malignant [6].

PGL3 syndrome (SDHC mutations) is commonly presented as a solitary head and neck paraganglioma. Furthermore, there is very low risk of malignant transformation [9].

PGL4 syndrome (SDHB mutations) is the second most common type of familial paraganglioma**.** SDHB acts as a tumor suppressor gene, mutations in SDHB are associated with dysregulation of the hypoxia pathway including over-expression of HIFα (Hypoxia-Inducible Factor alpha) and gene products such as VEGF (Vascular Endothelial Growth Factor). Most SDHB tumors are extra-adrenal. This syndrome provides a high risk of malignancy which has been estimated to range from 31%–71%. This mutation also confers susceptibility to other cancers such as gastrointestinal stromal tumors (GIST), papillary thyroid cancer, neuroblastoma and various types of renal cell carcinoma [9,10].

PGL5 syndrome (SDHA mutations) has been identified in only six patients [9].

Other associated AD hereditary syndromes are Multiple Endocrine Neoplasms Type 2 (MEN2), Neurofibromatosis 1 (NF1), Von-Hippel-Lindau (VHL) and Carney-Stratakis syndrome [5].

### Clinical presentation and imaging findings

CBTs diagnosis is performed by the combination of clinical and imaging findings. CBTs are rarely hyperfunctionant tumors. Therefore, they usually present as a painless neck mass with slow growth. At neck exploration, the “Fontaine sign” can be found, where the mass is mobile horizontally but not vertically and could be pulsatile.

An important aspect of CBTs evaluation is cranial nerves examination, as large CBTs may cause compression of lower cranial nerves (IX-XII), transient ischemic attacks and even strokes [1,3]. In case of hyperfunctional CBTs, the signs and symptoms are caused by the excess of catecholamines. Patients may present due to headaches, palpitations, swelling, flushing, hyperglycemia, fever, nausea, pallor, hypertension, arrhythmias, stroke or even anxiety that can lead to a myocardial infarction [11].

In terms of imaging, duplex ultrasound is the main diagnostic method since it confirms the anatomical relationship of the tumor to the carotid bifurcation and its vascularization(12). Besides duplex ultrasound, there are different imaging methods recommended for preoperative assessment as MRI, magnetic resonance angiography (MRA), computed tomography (CT), digital subtraction angiography (DSA) and computed tomography angiogram (CT angiogram). Characteristic findings on MRI are T2 “salt and pepper” imaging and lesions which are isointense on T1 and hyperintense on T2 [12].

On CT, CBTs characteristics are hypervascular masses located at the carotid bifurcation. CT angiography findings are an hypervascular mass with enlarged arteries, tumor blush, early draining veins and lyre sign (splaying of internal and external carotid arteries). Indium-111 octreotide, is a nuclear imaging study that identifies multicentric or metastatic paragangliomas and detects residual tumor after surgery [2,12].

Although the extent of the tumor is often demonstrated by other imaging studies such as CT and/or MRI, vascularity is best studied with DSA, it also helps to observe the displacement and compromise of blood vessels, as well as the adequacy of the intracranial circulation if internal carotid artery sacrifice is necessary [13], [14], [15]. In turn, it can reveal previously unsuspected synchronous paragangliomas. Nevertheless, selective arteriography is not a routine study in the diagnosis of carotid body paraganglioma, unless preoperative embolization is considered prior to surgery [13], [14], [15].

### Histopathologic findings

Macroscopic characteristics involve a tumor that rarely exceeds 6 cm in diameter and arises near or envelops the bifurcation of the common carotid artery. It has a macroscopic fleshy appearance and the tissue is red-pink to brown due to bleeding or fibrosis [5,16]. Histologically, the tumor consists of polygonal or spindle chief cells with eosinophilic and uniform cytoplasm arranged in small nests (Zellballen) surrounded by sustentacular cells. The nests are separated by a delicate fibrovascular stroma. The nuclei are round or oval and have prominent nucleoli [5,16]. The chief cells stain strongly for neuroendocrine markers, including chromogranin, synaptophysin, neuron-specific enolase, CD56 and CD57. Sustentacular cells are positive for the S-100 protein [16].

### Treatment and prognosis

The main treatment for CBTs is surgical resection of the tumor. The cure rate after complete resection of a benign carotid body tumor is 89% – 100%. Although conservative treatment can be used for asymptomatic patients, the vast majority of them will become symptomatic in the future. Shamblin, et al. developed a surgical classification system which predicts surgical morbidity. Shamblin classification divides the CBTs in three different groups based on operative notes and tumor relationship with vessels [17], [18], [19].

Group one are easily resectable tumors with minimal adherence to vessels. Group two tumors are partially surrounded by vessels and adhered to the adventitia. Finally, group three tumors are adherent and intimate with the carotid bifurcation. The third group of the Shamblin classification tumors are the most difficult to remove and present frequent neurological consequences after surgery [1,2].

CBTs present an extensive growth and may encase vital neurovascular structures in the neck, making surgical resection extremely challenging due to hypervascularity (with an average blood flow of 200 mL/g per minute) and possible significant blood loss [12]. For very vascular and large CBTs, embolization before surgery has demonstrated safer resection by decreasing vascularity and dimensions of the tumor. Occasionally, CBTs are unresectable prior to embolization, but shrink and become resectable [20, 21]. The goal of embolization is to obliterate selectively vascular structures after assessing which artery provides the tumor most blood supply [20,21].

Embolization of the tumor's main artery (usually the pharyngeal ascending artery) prior to surgery may help reduce bleeding and other complications associated with the removal of large tumors, facilitating resection [20,21]. A retrospective analysis of 35 patients revealed significant reduction of blood loss during surgery in patients with a preoperative embolization versus non embolized patients (mean 1,122 mL vs 2,769 mL, respectively) [22].

A consensus has not yet been established regarding the indications for preoperative arterial embolization [23,24]. Some criteria used by different authors include: size (typically >3cm), stage of the disease, class C and D jugular paragangliomas and scale of Shamblin III [15, 25, 26]. However, potential complications such as skin necrosis, blindness, cranial nerve deficit, stroke, and death must be considered, since embolization is an invasive and potentially dangerous procedure. The risk of these complications ranges from 0% – 13% [15,23].

The most probable etiologic factor related to the bilateral carotid body tumor presented in this case is related to high altitudes. Mexico City has an average altitude of 2,240 meters above sea level, causing mild hypoxemia and chronic stimulation of the carotid body [1]. The present case addresses a new strategy for preoperative management of a carotid body tumor. Due to significant fragility of the carotid vessels prior to surgical resection, a covered graft-stent was placed in the right common and internal carotid arteries (C1 segment). Carotid arteries were splinted and blood flow to the right external carotid artery was temporarily blocked. Therefore, reducing blood supply to the carotid body tumor in order to reduce its size and vascularity prior to surgical resection. After the procedure, the patient underwent an MRI and FLAIR sequence control in order to discard any ischemic lesion, finding no abnormalities and was followed-up two weeks after, finding no clinical alterations on physical examination nor image studies.

Resection of bilateral carotid body tumors can cause baroreflex failure syndrome, characterized by severe and constant hypertension during the first 24 to 72 hours after surgery, followed by labile hypertension and hypotension, headache, emotional instability and palpitations [27,28]. The ideal approach is to first perform surgical excision of the smallest tumor. If the vagus and hypoglossal nerves are functional, a contralateral surgery can be performed. In case of injury to these nerves, radiotherapy (RT) is appropriate for the opposite tumor due to risk of neurological deficit if the contralateral tumor is excised [29].

After surgery, patients should be monitored for neurological complications which are mainly temporary. Cerebrovascular events and mortality rates after surgery is less than 3%. The majority of patients are cured and present no recurrence after surgery (familial forms have and increased risk of recurrence and multifocality). In cases where the carotid body paraganglioma is malignant, it can be treated with RT even with metastasis, being the survival rate less than 50% in the next 10 years [2,12].

RT and chemotherapy can be alternatives when a surgical approach cannot be done but they are not as effective as surgery. Conventionally fractionated external beam RT or stereotactic body RT (SBRT) may be used for the treatment of benign non-catecholamine-secreting paragangliomas where resection represents an extensive sacrifice of critical vascular or neural structures, and for those with recurrent tumors after surgery. Although RT provides good long-term disease control, locally symptomatic lesions should be considered for surgery whenever anatomically feasible [30].

## Conclusion

Bilateral CBTs are rare neuroendocrine neoplasms usually detected in women with an average age of 43 years, with a female to male ratio around 8:1. When evaluating bilateral CBTs, etiologic factors such as genetic causes and patients living in high altitudes, should be assessed. In this case, we presented a novel therapeutic management prior to tumor resection in which the right common carotid and proximal internal carotid arteries were splinted by a covered graft-stent. The purpose was to reduce blood supply to the carotid body tumor in order to reduce its size and vascularity prior to surgical resection and provide structural protection to the carotid vessels, in order to ensure cerebral blood flow and perfusion during the procedure.

## Conflict of Interest

The authors declare no conflicts.

## Patient consent

The authors confirm that patient consent was obtained for the publication of this Case Report.
